# Six-Digit CPK and Mildly Affected Renal Function in McArdle Disease

**DOI:** 10.1155/2014/704398

**Published:** 2014-10-09

**Authors:** George Spyropoulos, Panagiotis Kratimenos, Andrew D. Mcinnes, Richard J. DeGroote, Ioannis Koutroulis

**Affiliations:** ^1^University of Iowa Children's Hospital, Iowa City, IA 52242, USA; ^2^St. Christopher's Hospital for Children, Drexel University College of Medicine, Philadelphia, PA 19134, USA; ^3^The Unterberg Children's Hospital at Monmouth Medical Center, Drexel University College of Medicine, Long Branch, NJ 07740, USA

## Abstract

A previously healthy, white 12-year-old girl presented with diffuse body aches and poor perfusion. She developed severe respiratory failure and marked rhabdomyolysis and was mechanically ventilated. Although her CPK peaked at 500,000 IU/L, her renal function was mildly affected and her creatinine did not exceed the 0.8 mg/dL. The rhabdomyolysis was gradually resolved following aggressive fluid hydration. The patient did not require dialysis and made a complete recovery. Genetic studies revealed the diagnosis of McArdle disease.

## 1. Introduction

Rhabdomyolysis is the result of muscle breakdown which could eventually lead to serious complications such as acute renal injury and failure with some patients even requiring renal transplant. Viral myositis and trauma are the leading causes of rhabdomyolysis followed by dermatomyositis, drug-induced rhabdomyolysis, and metabolic disorders [1]. McArdle disease is a common glycogen disorder of the muscle tissue and is characterized by childhood onset exercise intolerance with 50% of the patients experiencing both rhabdomyolysis and myoglobinuria, while a number of affected individuals are subjected to dialysis due to subsequent renal failure [2, 3].

We report a case of a teenager girl who presented in shock and severe rhabdomyolysis requiring a prolonged hospitalization with mechanical ventilation in the Pediatric Intensive Care Unit of The Unterberg Children's Hospital at Monmouth Medical Center, Long Branch, NJ. Despite her extremely elevated creatinine phosphokinase (CPK) levels (up to 500.000 IU/L), she responded adequately to prompt fluid resuscitation, urine alkalization, and low dose dopamine infusion. The patient recovered without the need for renal replacement therapy. Genetic studies revealed the diagnosis of McArdle disease.

## 2. Case Report

The patient was a previously healthy 12-year-old female who presented to the Emergency Department (ED) one day prior to admission with a 3-day history of malaise, decreased appetite, bilateral lower extremity muscle aches and diffuse dull, and nonradiating abdominal pain. There was no history of fever, cough, vomiting, diarrhea, or dysuria and her last menstrual period was 1 month ago. She was hydrated with a normal saline bolus, received ibuprofen for pain relief, and was discharged home asymptomatic. She returned to the ED the very next day where she was diaphoretic and cold reporting that the lower extremity muscle aches and fatigue had worsened.

Her temperature was 96.5 F (rectal), blood pressure was 135/83 mmHg, heart rate was 123/min, respiratory rate was 26/min, and her capillary refill was greater than 3 sec. She was alert and oriented to time, place, and person. On physical examination, she had cold extremities with dry oral mucosa and appeared in no respiratory distress. Bilateral lower extremity muscle tenderness to palpation was noticed and the rest of the physical exam was unremarkable.

Her laboratory results from her first ED visit included hemoglobin of 17.5 mg/dL, hematocrit of 52.1%, and white blood cell count 10.9 M/mm^3^ with 76% neutrophils, 12% lymphocytes, 12% monocytes, 0% basophils, and 0% eosinophils. Her CPK was 184 IU/L, Cr was 0,74 mg/dL, and blood urea nitrogen (BUN) was 17 IU/L. The poor perfusion in the absence of gastrointestinal symptoms raised suspicion for infection and she was treated with IV ceftriaxone and vancomycin.

**Figure 1:**
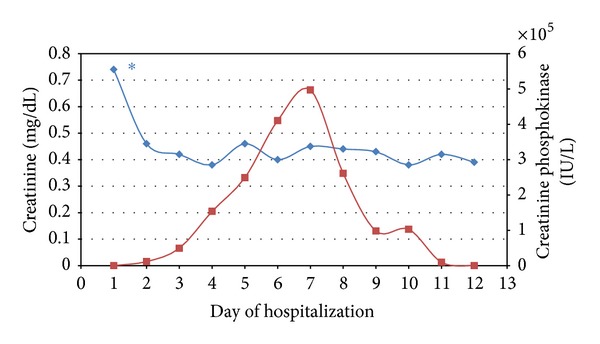
Creatinine levels (blue line) and creatinine phosphokinase levels (red line) during hospitalization. *Initiation of fluid resuscitation and urine alkalization.

She was admitted to the Pediatric Intensive Unit (PICU) for fluid resuscitation and observation. Her perfusion improved after a dose of 40 mL/kg of bolus hydration with normal saline. Initially, she remained hemodynamically stable, but the body aches recurred. On day 2 of admission, she complained of shortness of breath and chest pain and had decreased responsiveness and shallow respirations. Her blood pressure increased to 160/140 mmHg and her Glasgow Coma Scale (GCS) score was 6 (Eye 1 + Verbal 1 + Motor 4). Emergent rapid sequence intubation was performed and she was on pressure regulated volume control (PRVC) ventilation. Magnetic resonance imaging (MRI) of the brain ruled out increased intracranial pressure. On the following days, her CPK continued to rise gradually up to 500,000 IU/L while she was receiving double maintenance IV hydration with normal saline and continuous infusion of dopamine at 3 mcg/kg/min. Her renal function remained mildly affected, as shown by her Cr levels (Figure 1), and she did not require renal replacement therapy. She remained mechanically ventilated for a total of 4 days and she gradually became asymptomatic. The CPK started to decrease until she was discharged from the hospital. An extensive genetic workup was performed and she was diagnosed with McArdle disease.

## 3. Discussion

McArdle disease (glycogen storage disease Type V; MD) is a disorder of the muscle metabolism. Numerous mutations of the PYGM gene have been identified to be linked to the disease, with the nonsense mutation p.R50X being the most common [3]. Prevalence of the disease is estimated to be 1 in 100.000 in some areas of the US [4]. As in our patient, childhood onset exercise intolerance manifesting as fatigue, myalgia, stiffness, or weakness is the main clinical feature of MD. Diagnosis requires a high index of suspicion. CPK remains elevated even at rest. The diagnostic tests that are commonly used are serum CPK levels, ischemic forearm exercise test, muscle biopsy, and molecular genetic studies [5, 6]. Our patient's diagnosis was based upon the clinical symptoms, the extremely high levels of CPK (up to 500.000 UI/L), and the acute renal failure (ARF) caused by the severe rhabdomyolysis. There is no definitive treatment for MD, but gene replacement therapies, high protein diet, sucrose diet, supplementation of Vitamin B6, oral creatinine, and moderate aerobic exercise all have been suggested in the management of the disease [7–11]. However, it is crucial to rapidly recognize rhabdomyolysis and intervene appropriately in order to prevent acute renal injury.

Rhabdomyolysis is the result of skeletal muscle cell breakdown, occurring when normal cell contents, such as creatine phosphokinase (CPK), myoglobin, phosphorus, and potassium, are released into the bloodstream. The most significant complication of this gross spillage is kidney damage [12, 13]. Pediatric patients are at risk for rhabdomyolysis due to various causes, with viral myositis and trauma being the most common. The rate of the associated ARF is estimated to be around 5% [1].

Laboratory diagnosis of rhabdomyolysis is based on the determination of plasma CPK, which is the most sensitive marker [14–16]. High CPK values upon admission, peak CPK values, and slower decline of serum CPK values are associated with ARF [17]. Although there is no established cut-off threshold, a concentration 5 to 10 times the upper limit of the normal reference range (i.e., 500–1000 U/L) is commonly used and concentration > 5000 U/L is closely related with development of acute kidney injury and recommendations suggest close monitoring of renal function [14–16]. Fernandez et al. in a recent study showed that the most reliable predictor for ARF and need for dialysis is Cr > 1.7 despite the peak of CPK; however, the mean peak of CPK in their study was 43578 UI/L which is more than ten times lower than the CPK peak of our patient [18]. Myoglobin serum and urine concentrations might be useful in the early stages but are not essential for the diagnosis. Acute renal failure is the most common complication, but cardiac arrest and compartment syndrome may also be caused by the severe hyperkalemia and hypocalcemia that occur [14–16]. Although the exact mechanism by which rhabdomyolysis causes renal failure is unknown, renal vasoconstriction/hypoperfusion, renal tubular obstruction secondary to cast formation, and myoglobin-mediated tubular cytotoxicity are the proposed mechanisms [12, 13]. ARF and mortality in pediatrics patients are linked, with the odds of mortality increasing with RIFLE (risk, injury, failure, loss, end stage kidney disease) score [19].

Early and aggressive volume resuscitation is the mainstay for preventing and treating acute kidney injury (AKI) in rhabdomyolysis. Alkalization of the urine and forced diuresis with mannitol and loop diuretics has also been used in the prevention and management of AKI. Finally in severe cases of AKI or when initial treatment fails, renal replacement therapy may be required [12, 14].

Our patient experienced severe rhabdomyolysis as indicated by her extremely high CPK level. Although unclear, we hypothesize that our patient's final good outcome resulted from the early recognition and very prompt and simultaneous administration of intravenous fluids, nephroprotective dopamine infusion, and urine alkalization, particularly prior to the peak CPK rise.

We report the first case of rhabdomyolysis in a child with six-digit CPK who did not require dialysis.
